# In Silico Studies on Triterpenoid Saponins Permeation through the Blood–Brain Barrier Combined with Postmortem Research on the Brain Tissues of Mice Affected by Astragaloside IV Administration

**DOI:** 10.3390/ijms21072534

**Published:** 2020-04-05

**Authors:** Katarzyna Stępnik, Wirginia Kukula-Koch

**Affiliations:** 1 Department of Physical Chemistry, Institute of Chemical Sciences, Faculty of Chemistry, Maria Curie–Sklodowska University in Lublin, Pl. M. Curie-Sklodowskiej 3, 20-031 Lublin, Poland; 2 Chair and Department of Pharmacognosy, Medical University of Lublin, ul. Chodzki 1, 20-093 Lublin, Poland; virginia.kukula@gmail.com

**Keywords:** QSAR, triterpenoid saponins, computational studies, brain tissue, astragaloside, Astragalus, milkvetch, *Fabaceae*

## Abstract

As the number of central nervous system (CNS) drug candidates is constantly growing, there is a strong need for precise a priori prediction of whether an administered compound is able to cross the blood–brain barrier (BBB). The aim of this study was to evaluate the ability to cross the BBB of triterpenoid saponins occurring in *Astragalus mongholicus* roots. The research was carried out using in silico methods combined with postmortem studies on the brain tissues of mice treated with isolated astragaloside IV (AIV). Firstly, to estimate the ability to cross the BBB by the tested saponins, new quantitative structure–activity relationship (QSAR) models were established. The reliability and predictability of the model based on the values of the blood–brain barrier penetration descriptor (logBB), the difference between the *n*-octanol/water and cyclohexane/water logP (ΔlogP), the logarithm of n-octanol/water partition coefficient (logP_ow_), and the excess molar refraction (E) were both confirmed using the applicability domain (AD). The critical leverage value h* was found to be 0.128. The relationships between the standardized residuals and the leverages were investigated here. The application of an in vitro acetylcholinesterase-inhibition test showed that AIV can be recognized as the strongest inhibitor among the tested compounds. Therefore, it was isolated for the postmortem studies on brain tissues and blood using semi-preparative HPLC with the mobile phase composed of water, methanol, and ethyl acetate (1.7:2.1:16.2 *v*/*v*/*v*). The results of the postmortem studies on the brain tissues show a regular dependence of the final concentration of AIV in the analyzed brain samples of animals treated with 12.5 and 25 mg/kg b.w. of AIV (0.00012299 and 0.0002306 mg, respectively, per one brain). Moreover, the AIV logBB value was experimentally determined and found to be equal to 0.49 ± 0.03.

## 1. Introduction

The blood–brain barrier (BBB) term is used to describe the unique properties of the microvasculature of the central nervous system (CNS) [1]. The endothelial cells of brain microvasculature are the anatomical components of the BBB. They form tight junctions, which, along with pericytes, astrocytes, oligodendrocytes, microglia, and neurons, construct the neurovascular unit [2]. Pericytes, being embedded in the basement membrane of brain capillaries, play a key role in the development of cerebral microcirculation [3,4]. Some previous studies have shown that, for instance, pericyte deficiency leads to brain vascular damage and can also be responsible for BBB breakdown [2,3,4].

The blood–brain barrier forms a dynamic interface between blood and the brain and is a diffusion barrier that is essential for the normal functioning of the central nervous system [5]. The endothelial cells of the BBB regulate CNS homeostasis and protect the CNS from toxins, pathogens, inflammation, injuries, and diseases [1]. Despite the protective nature of the blood–brain barrier on the CNS, the inability of substances to cross the BBB is a key factor that should be taken into account in the early stages of drug discovery processes [6]. The BBB is present along the vasculature of the brain except for the circumventricular organs where the blood vessels of the brain have fenestrations that permit the diffusion of blood-borne molecules across vessel walls [5,7]. The BBB is formed by endothelial cells that line cerebral microvessels [7].

The passive passage of molecules across the endothelium cells of the BBB can occur through the paracellular (between adjacent cells) or transcellular (through the cells) pathways [8]. The paracellular pathway can only allow small, usually hydrophilic, solutes. These molecules and/or ions simply diffuse between adjacent cells down their concentration gradient [9,10]. The transcellular pathway involves different mechanisms including the passive diffusion of lipophilic compounds, receptor-mediated shuttling, and transcytosis [7,11]. Some molecules such as oxygen, CO_2_, alcohol, and steroid hormones penetrate transcellularly by dissolving in their lipid plasma membrane [12,13]. They can pass the BBB freely by diffusion [14]. For almost all other substances, including essential materials such as glucose and amino acids, transport proteins (carriers), which are specific receptor–mediated or vesicular mechanisms (adsorptive transcytosis), are required to pass the BBB [12]. This indicates that hydrophilic molecules may enter the brain through specific transport mechanisms [15].

The experimental determination of BBB permeability is usually time consuming and expensive and requires complex techniques. Therefore, it seems to be very difficult [16,17,18]. However, modern methods do exist, including computational and non-cell-based in vitro approaches. Among them Parallel Artificial Membrane Permeability Assays (PAMPAs) and quantitative structure–activity relationship (QSAR) analysis both provide fast analysis of BBB permeation, which is of particular importance in the early stages of drug discovery processes [19,20,21]. In the area of the CNS, there are widely used QSAR predictive models [22] based on various combinations of physicochemical parameters. In Table 1, some of the previously reported QSAR models are compared with the newly established models.

Most of the recognized QSAR models prove that there is a relationship between molecule transport across the blood–brain barrier and the steric, lipophilic, and electronic characteristics of a molecule, which is obviously consistent with the Hansch approach [35,36,37,38,39].

We established new models here to estimate the BBB penetration of astragalosides which are triterpenoid saponins, composed of triterpene aglycones joined with various sugar moieties. They are generally predominant in cultivated crops, mainly in legumes such as soybeans, beans, peas, *Lucerne*, etc. as well as in alliums, tea, spinach, sugar beet, quinoa, liquorice, sunflower, horse chestnut, and ginseng [40].

Astragalosides commonly exist in the roots of different types of milkvetch, including *Astragalus membranaceus* (Fisch.) Bunge and *Astragalus mongholicus* Bunge (Fabaceae). *Astragali radix* being dried roots of the above-mentioned plants is the name of an herbal drug frequently used in traditional Chinese medicine, however, also recognized practically all over the world [41]. It owes its vast applicability due to a wide spectrum of action on living organisms.

The tested saponins possess various biological activities, among which the antioxidant, antifungal, molluscicidal, anti-inflammatory, antitumor, and antiviral properties are most widely described [42]. Astragalosides (especially astragaloside IV) show immunostimulant, anti-perspirant, antidiarrheal, anti-diabetic, and tonic properties, among others [43]. Moreover, they are characterized by their anti-cancer effect on lung, gastric, breast, and colorectal cancer (in vitro tests) [44,45,46,47]; anti-photoaging effects [48]; and influence on the cardiovascular and nervous systems, the metabolism of collagen, and the immune system [49].

The aim of this study was to evaluate the ability of triterpenoid saponins occurring in *Astragali radix* to cross the BBB based on a QSAR methodology combined with postmortem studies on the brain tissues of mice. QSAR was investigated here using linear free energy relationships (LFER) descriptors [35,36,37,38,39], as well as steric, lipophilic, and electronic parameters. In this investigation, the relationships between the logBB values and various partition indices were examined to compare their possible effectiveness in describing BBB passage, e.g., in Equation (2), we combine the logBB values with the hydrogen bond ΔlogP value, the lipophilic logP_ow_ value, and the excess molar refraction E. Moreover, we experimentally determined the logBB value for the most active saponin (AIV) in mice. This is the first time such an experiment has been performed. All planned studies were carried out to show the path from in silico modeling to the postmortem determination of both the logBB value and the concentration of the most neuroactive components of *Astragalus* roots in the brain tissues of mice.

## 2. Results

### 2.1. Division of the Dataset for the Computational Studies

The dataset used here includes 47 chemically diverse compounds (most of them with the corresponding experimentally determined logBB values), which were taken from the literature [50], including the tested triterpenoid saponins. The chemical structures of the investigated saponins are presented in Table 2. The dataset was separately divided into training and test sets in a random manner for each of the newly constructed models. In total, a random division of the whole dataset was made several times. The lowest value of the mean square error of the leave-ten-out cross-validation (i.e., the adjusted mean square error of leave-ten-out cross-validation (adjusted MSECV)) process decided between inclusion in either the training or test set. Among the tested substances, 10 were chosen as the test set, whereas 30 compounds were selected as a training set and then were used to establish new QSAR models. The tested saponins were external for the models, meaning that they were not used to develop any of the models. Moreover, the self-contained test set, comprised of seven substances, was used for the external validation. The predictive potency of newly constructed QSAR models was estimated by the leave-ten-out (LTO) cross-validation procedure. The coefficient of determination (R^2^), root-mean-square error (RMSE), root-mean-square error of leave-ten-out cross-validation (RMSECV), and predicted residual sum of squares (PRESS) statistical parameters were obtained. The QSAR models were based on the multiple linear regression (MLR) methodology with the backward elimination of variables in order to limit the differences between the actual and the estimated BBB values. Many attempts were made to obtain the best relationships between the logBB values and various physicochemical descriptors. The best models were selected based on the analysis of variance using the adjusted sum of squares (adjusted SS), adjusted mean square errors (adjusted MSE), standard errors (SE), variance inflation factors (VIF), R^2^ values, *p*-values, T-values, and the Fisher criterion (F-values) parameters. Then, to evaluate the reliability of the QSAR models, the applicability domain (AD) was applied.

### 2.2. BBB Descriptors Calculated in Silico

At the stage of the in silico computational studies, the most important pharmacokinetic descriptors of the brain for the tested saponins were calculated, i.e., the blood–brain barrier penetration descriptor (logBB), the permeability–surface area product (PS) usually given as a logPS value, the brain/plasma equilibration rate (log(PS_Fubrain_)), the fraction unbound in plasma (F_u_), and the fraction unbound in the brain (F_b_) (Table 3).

It was observed that a decrease in the BBB permeability–surface area product (PS) or the fraction unbound in the brain (F_b_) prolonged the time to reach equilibrium in the brain [51]. However, this time value did not change when PS decreased and F_b_ increased or, inversely, when PS_Fubrain_ was kept constant. Therefore, compounds having similar PS_Fubrain_ values should exhibit a similar time to reach equilibrium, although they may have a much different PS value.

In our experiment, the logBB values, predicted for substances I–IV, were greater than 0, with average logPS values equal to −4.5. These values reveal that compounds I–IV can cross the BBB and that they may have neuroactive potential. In contrast, astragalosides V–VII have a logBB value <−2. At the same time, substances V–VII have a logPS value <−5. This indicates that those compounds are not permeable through the BBB, even though the in silico estimated values of the fraction unbound in plasma are relatively high. Accordingly, astragalosides V–VII were neglected in further procedures, i.e., in both the in vitro and postmortem studies.

### 2.3. QSAR Studies for the BBB Permeation

To predict the ability of the seven tested saponins which naturally occur in the roots of *Astragalus mongholicus* to cross the blood–brain barrier, new QSAR models were generated using experimentally obtained logBB values for 40 other molecules that have been reported in the literature [50] (Table S1). Therefore, the studied compound group consisted of seven substances (Table 2), while 40 compounds were selected to establish the QSAR models (Table S1). In the QSAR methodology, many physicochemical descriptors are used to predict various biological activities. According to the Hansch approach, the most important parameters governing transport and drug–receptor interaction are the steric, electronic, and lipophilic characteristics of molecules [35].

Another commonly used approach is the linear free energy relationship (LFER), suggested by Abraham, which is based on parameters such as hydrogen-bond acidity (A) and basicity (B), polarizability (S), molar refraction (E), and the McGowan volume (V) of a solute. In our research, we used both of the above-mentioned theories. Therefore, the most important physicochemical descriptors, as well as the LFER parameters, were calculated and are presented here in Table 4 (ACD/Percepta software). On the basis of the LFER parameters, the following model (Equation (1)) was generated:

logBB = −0.118 − 0.11 A − 1.174 B − 0.176 S − 0.242 E + 1.195 V(1)

In the above equation, *n* = 40, R^2^_CV_ = 80.80%, R^2^_pred_= 76.80%, and S = 0.432, where *n* is the number of compounds, R^2^_CV_ is the cross-validated coefficient of determination, R^2^_pred_ is the predicted R^2^ value, and S is the standard deviation.

In the next model (Equation (2)), we decided to correlate BBB penetration with the lipophilic properties (logP_ow_), excess molar refraction (E), and hydrogen-bonding potential, expressed as the difference between the *n*-octanol/water and cyclohexane/water logP values (ΔlogP) [52,53,54]. Thus, we built the following model:

logBB = −0.114 − 0.098 ΔlogP + 0.278 logP_ow_ + 0.218 E (2)

In the above equation, *n* = 40, R^2^_CV_ = 78.25%, R^2^_pred_ = 74.02%, and S = 0.436.

**Figure 1:**
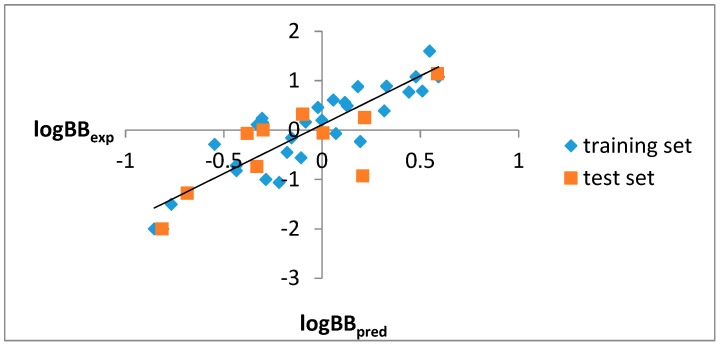
Experimental logBB values (logBB_exp_) [50] vs. in silico logBB values, predicted on the basis of Equation (2) (logBB_pred_).

On the basis of Equation (2), the logBB values were calculated (logBB_pred_) and then they were correlated and compared with the experimental logBB values (logBB_exp_), taking into account the training and the test sets (Figure 1).

According to Figure 1, it can be stated that both tools used, i.e., the ACD/Percepta software and the newly established QSAR (Equation (2)), can be recognized as good predictive models for assessing the ability of the tested compounds to penetrate through the blood–brain barrier.

### 2.4. Applicability Domain

There are regulations related to the use of alternative methods to in vivo ones, which are used especially in the initial phases of research on various compounds. The main aim of the European Centre for the Validation of Alternative Methods (ECVAM) is to promote alternative methods which *reduce, refine,* and *replace* the use of laboratory animals. According to the Organisation for Economic Co-operation and Development (OECD) and the European Commission guidance, general principles exist for QSAR validation [55]. One of them states that “a (Q)SAR should be associated with a defined domain of applicability”. The applicability domain (AD) should be understood as the response and chemical structure space in which the model makes predictions with a given reliability [56]. There is a need to establish the scope and limitations of a model based on the physicochemical and response information in the model training set. In our case, the response space concerns the BBB permeation of compounds. The AD was evaluated to confirm the reliability and predictability of the model expressed in Equation (2). Different approaches have been developed for statistically-based QSARs. In our experiment, we used a distance-based method, which can be used to separate regions of varying density by imposing cut-off values. The distance from a query data point to a dataset was calculated via this approach. If there is a criterion for the distance to be below a defined threshold, the decision as to whether a data point is close to the dataset can be made [56].

The model space can be represented by the descriptor matrix (X), which is the two-dimensional matrix comprised of *n* chemicals (rows) and *k* variables (columns). A measure of the distance of the chemical from the centroid of X can define the leverage of a chemical (h_i_). The leverages of all chemicals in the dataset were generated here by manipulating X according to the following equation [56] (Equation (3)):

h_i_ = (X^T^X)^−1^x_i_^T^ (3)

where *i* = 1 to *n*, x_i_ is the row vector of the descriptors, T is the matrix/vector transposed, and i and X are the variable matrices deduced from the training set variable values.

The critical leverage value (h*) can be generally obtained using the following equation (Equation (4)):

h* = 3(k + 1)/*n* (4)

where *k* is the number of predictor variables and *n* is the number of training compounds [56].

**Figure 2:**
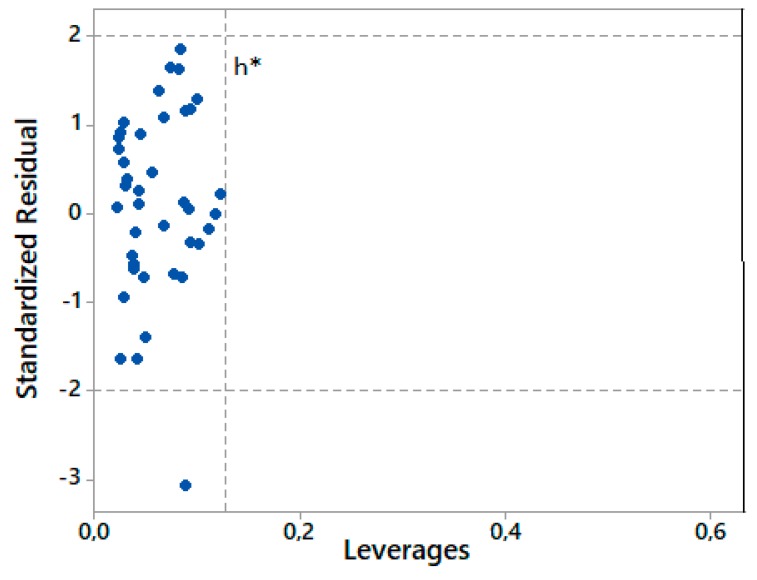
Williams plot of the new QSAR model (Equation (2)).

To identify both the chemicals that are outside the AD and the leverage points which could destabilize the model, the Williams plot can be used. The plot is presented in Figure 2. The standardized residual equals the value of a residual divided by an estimate of its standard deviation.

**Figure 3:**
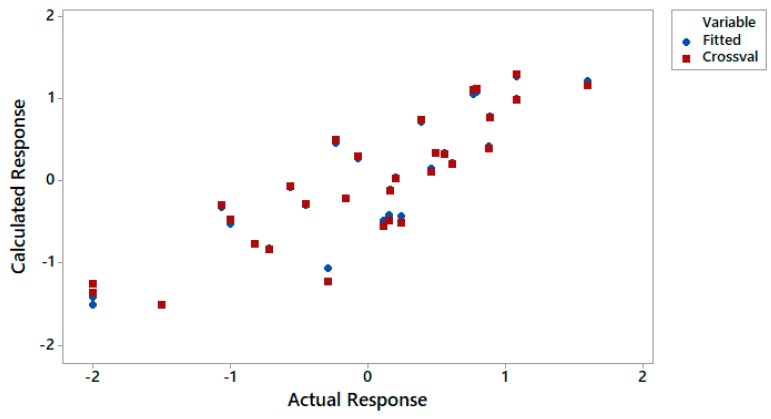
Scatterplot of the fitted and cross-validated responses versus the actual responses.

The h* value calculated for our QSAR model (Equation (2)) was equal to 0.128. It is assumed that a compound where h_i_ > h* has a great impact on regression occurrence; therefore, it may be excluded from the AD. It is assumed that substances with a standardized residual greater than 3SD (standard deviation) units may recognized as outliers, while chemicals with a leverage value higher than h* are recognized as influential or as high-leverage chemicals [57]. In the process of developing the applicability domain, substances that were not previously used to build the QSAR model were used. In other words, the applicability of model was valued here, indicating how well it is able to predict the end-point values of the compounds that did not develop the correlation. In our case, such substances were triterpenoid saponins. As shown in Figure 2, all substances, including triterpenoid saponins, were within the AD. Moreover, the correlation between the fitted and cross-validated responses versus the actual responses is presented in Figure 3. It was found that there were no considerable differences between the fitted and the cross-validates values. Based on the results in Figure 2 and Figure 3, it can be proved that our QSAR model is reliable within the applicability domain in which was developed.

### 2.5. Acetylcholinesterase-Inhibition Activity Test

The inhibition of acetylcholinesterase (AChE) is considered to be a promising strategy for the treatment of neurodegenerative diseases. The main role of AChE is the termination of nerve impulse transmission at cholinergic synapses via the rapid hydrolysis of acetylcholine (ACh). An increase in AChE levels can be observed in elderly patients, leading to the excessive degradation of acetylcholine before it binds to the receptors in the postsynaptic membrane. A very quick degradation of acetylcholine, especially when levels are already significantly reduced at an elderly age, causes neuronal signal transmission to be disturbed. In view of the above, AChE inhibitors are seen as drugs that can effectively improve the cognitive functions of the brain. Compounds from this group are commonly seen as primary drugs for treating dementia. However, due to various adverse effects of synthetic drugs used in the therapy of memory disorders and their pharmacokinetics, new AChE inhibitors are constantly sought, mainly from natural sources [58].

**Figure 4:**
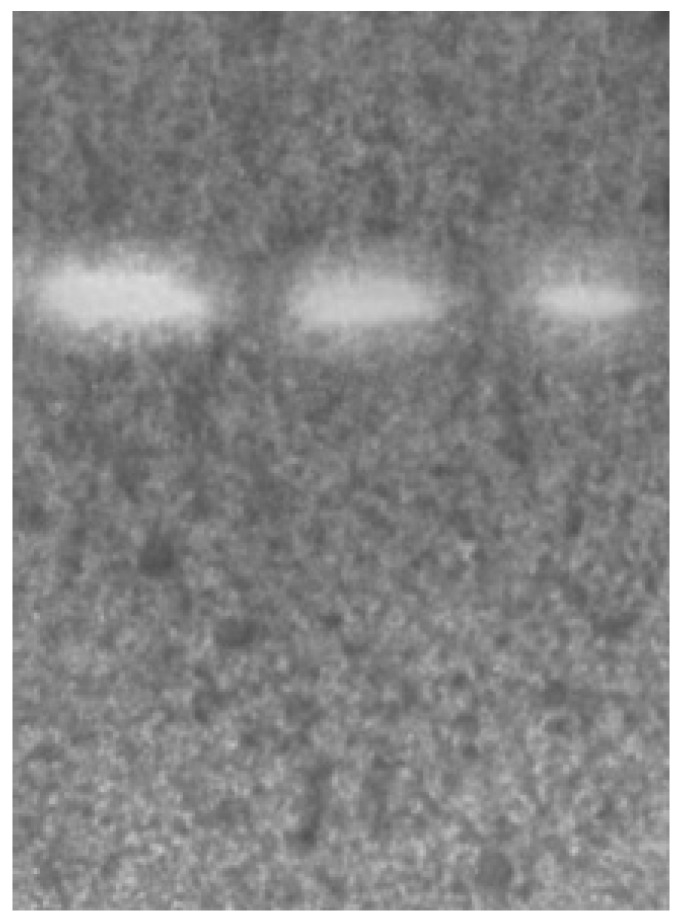
The chromatogram of the acetylcholinesterase inhibition activity in a thin layer chromatography (TLC)-bioautography based assay obtained for three astragalosides, namely, astragalosides IV (left spot), III (middle spot), and I (right spot).

To confirm the effectiveness of the tested compounds on the CNS, an acetylcholinesterase inhibition activity test can be performed using in vitro or in vivo (postmortem) tests. The former approach has commonly been used by numerous researchers, and the shape of the applied methodology varies, depending on the substrate or color developer [59,60,61]. Thin layer chromatography (TLC)-bioautography helps to clearly indicate the potential inhibitory action against AChE. In the applied methodology, the developed TLC plates were treated with an enzyme itself (AChE), and, by using this technique, active AChE inhibitors appeared as bright spots on a purple background. The intensity of the color and size of the spots in direct comparison (on the same TLC plate) with a reference compound allows the determination of the strength of the tested compound [59]. Analyzing the in silico obtained parameters (Table 3), three saponins which can cross the BBB most easily and therefore require the shortest time to achieve brain equilibrium (astragalosides I, III and IV) were further examined. As mentioned above, the in silico estimated BBB data show that astragalosides V–VII are not permeable across the BBB, and, therefore, they were excluded from the further research presented in the manuscript, whereas among the tested compounds that can cross the BBB (i.e., astragalosides I–IV), astragaloside II demonstrates the highest values of unbound fractions in both the brain and plasma and the lowest logBB value. As this compound requires the longest time to reach brain equilibrium, it was excluded from the studies on AChE inhibition activity. In Figure 4, the chromatogram of the AChE inhibition properties is presented. The white areas on the TLC chromatogram indicate the inhibitory activity of the tested astragalosides against acetylcholinesterase.

The chromatogram shows that AIV delivers the highest peak area, found using the ImageJ program (peak area of 7451 u), followed by AI (peak area of 4113 u), and AIII (peak area of 6556 u), and, therefore, it can be recognized as the most neuroactive compound among the tested compounds (Figure 4). The results of in silico and in vitro studies allow astragaloside IV to be selected as the most promising compound with potential procognitive properties. Therefore, the authors have planned its isolation from aqueous methanolic extract of *Astragalus mongholicus* roots.

### 2.6. Isolation of Astragaloside IV

**Figure 5:**
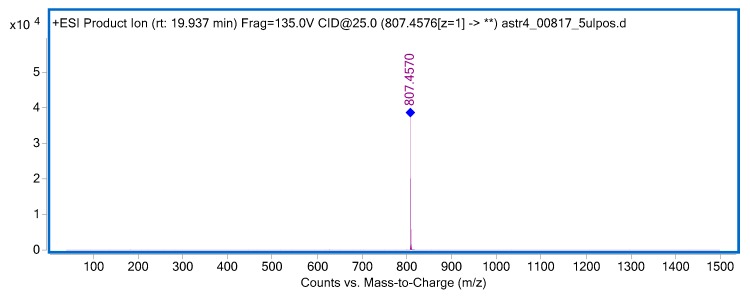
The MS/MS spectrum of astragaloside IV, recorded in the positive ionization mode and at a collision energy of 25V.

**Figure 6:**
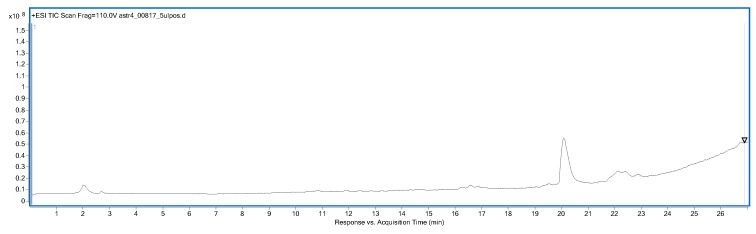
The total ion current (TIC) chromatogram of astragaloside IV, recorded in the positive ionization mode.

The isolation of astragaloside IV from aqueous methanolic extract of the roots was performed by semi-preparative HPLC chromatography. For this purpose, 52 fractions were collected in a single-cycle separation process. The compositions of the obtained fractions were analyzed using high performance liquid chromatography, coupled with mass spectrometry (HPLC-MS) to provide high-resolution qualitative data. The analysis confirmed the presence of high purity astragaloside IV in the tested fractions (Figure 5 and Figure 6) and those rich in the saponin of interest were collected together, evaporated to dryness under reduced pressure at 40 °C, and then used in the in vivo studies.

### 2.7. Postmortem Studies

Astragaloside IV was traced in the brain tissues and plasma of mice at two doses, namely, 12.5 and 25 mg/kg b.w. i.p. Before the LC-MS analysis, a thorough investigation of optimal chromatographic and spectrometric conditions was performed to select the settings that provide the highest response to the tested astragalosides, as described Section 4.6. The calibration curve of astragaloside IV obtained for the standard solutions was as follows: y = 1,052,449x + 28,518,981. The linearity of the graph was noted as R² = 0.9866 and was found to be precise enough to carry out quantitative studies. This slight decrease in the R² value was influenced by the wide range of concentrations analyzed in the study and was strongly influenced by the lowest concentrations of AIV. The limit of detection (LOD) values for this compound in the prepared method were calculated to be 5 µg/mL.

The presence of AIV was noted in the brain samples for the two groups of mice tested. The results show a regular dependence of the final concentration in the analyzed brain samples of animals treated with 12.5 and 25 mg/kg b.w. In these two groups, the concentration of astragaloside IV was calculated to be an average of 0.00012299 and 0.0002306 mg, per brain, respectively.

### 2.8. LogBB Determination

The blood–brain (BB) distribution is a measure which is defined as the brain/blood concentration. BBB penetration is frequently shown as logBB, which is the logarithm of the ratio between the brain and the blood concentration of tested substances [22,23]. The blood–brain distribution shows the suitability of a molecule for being a potential drug for the central nervous system [24], and, therefore, the ability of a molecule to penetrate the BBB is one of the biopharmaceutical properties that is essential in drug design. To be used as therapeutic agents, potential neuroactive compounds, which have to interact with their molecular targets in the CNS, must cross the BBB. To avoid side effects, peripherally acting agents should not cross the BBB simultaneously. In both cases, the BBB permeability of the molecules must be known [62].

The logBB value was calculated here based on the experimentally obtained concentrations of astragaloside IV in the brains and plasma of mice after the analyses of the two groups of animals that received 12.5 and 25 mg/kg b.w. of astragaloside IV. The obtained collective value of the logBB is equal to 0.49 ± 0.03 here.

**Figure 7:**
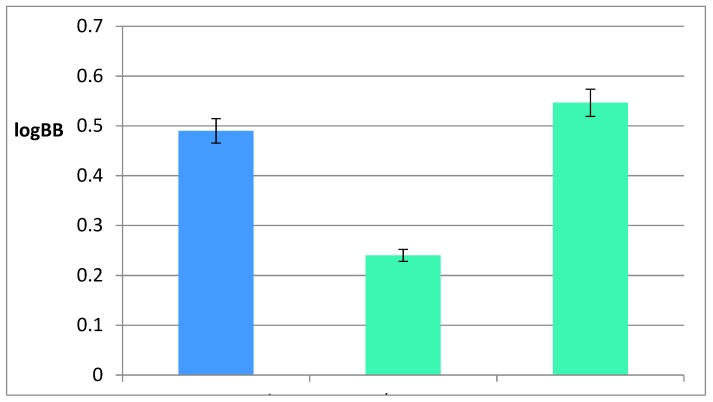
The comparison between the logBB values obtained experimentally, computed using ACD/Percepta software, and those calculated by the newly established model (Equation (2)).

Moreover, a comparison of logBB values for AIV predicted by our model with the values experimentally obtained and those determined using ACD/Percepta software has been made (Figure 7). The closest logBB value to the value obtained experimentally for the mouse brain tissues is that obtained from the Equation (2) (the absolute error is equal to 0.056). Therefore, it can be seen that the second model corresponds to the value obtained in vivo. Based on the analysis of variance (Table 5), Equation (1) was not used for further consideration.

## 3. Discussion

### 3.1. Computational Studies on BBB Permeation

To predict the BBB permeation of a substance, traditional statistical approaches are widely used, e.g., multiple linear regression (MLR), partial least square (PLS) methods, and linear discriminant analysis (LDA) [22,63,64,65]. The most frequently used in silico models of blood–brain barrier penetration are based on quantitative structure–activity relationships (QSAR); however, they are confined by the restricted accessibility of high-quality in vivo data in the early stages of the drug discovery process. Nevertheless, in silico studies are recognized to be useful in the preliminary assessment of BBB permeability, particularly due to their low costs and high efficiency [66].

The first paper that considered brain penetration analysis using computational modeling was published in 1988 [24], besides several previous reports on the use of various physicochemical properties of molecules in describing brain penetration. In this respect, the proton dissociation constant, partition coefficients measured in different solvent systems (heptane/water, benzene/water, and chloroform/water), and molecular size were recognized as being the most similar parameters to the partitioning characteristics of the BBB [67,68,69]. Young [24] derived a model based on a number of physicochemical parameters combining the ability to cross the BBB with a high level of H_2_ receptor histamine antagonist activity. He introduced numerous partition coefficients into his models, expressed as the logP value of different solvent systems, including *n*-octanol/water, chloroform/water, and cyclohexane/water, among others. The difference between *n*-octanol/water and cyclohexane/water logP values (ΔlogP) was first introduced by Seiler [52] and is related to the overall hydrogen-bonding ability of a molecule. This is because the hydroxyl group present in octanol is capable of forming hydrogen bonds, while cyclohexane, as a pure hydrocarbon, cannot.

In our experiment, new QSAR models based on the LFER parameters (Equation (1)) were used, as well as the excess molar refraction (E), the *n*-octanol/water logP value (logP_ow_), and the ΔlogP value (Equation (2)). Both the predictability and applicability of the model expressed in Equation (2) have been confirmed. As mentioned above, this model is reliable within the applicability domain in which it was developed.

#### 3.1.1. QSAR Based on LFER Parameters and Physicochemical Descriptors

The linear free energy relationship (LFER) of Abraham is used to characterize many biological and physicochemical processes, since the biological activities of solutes are based on the same basic intermolecular interaction forces, such as hydrophobic, electronic, and steric effects, as well as hydrogen bonds. The LFER is used to study human intestinal absorption [70,71], permeation and distribution across the BBB [72,73], and human skin permeation and partitioning [74]. The general LFER equation originally employed by Abraham et al. [70,71,72,73,74,75] was phrased as follows (Equation (5)):

SP = c + vV + sS + bB + aA + eE(5)

where SP is the dependent solute property in a given system, typically expressed as a logBB value, whereas the independent variables are solute descriptors, V is the solute McGowan volume in the units of cm^3^ mol^−1^/100, S is the polarizability/dipolarity, B is the overall hydrogen-bond basicity, A is the overall hydrogen-bond acidity, and E is the excess molar refraction. The coefficients v, s, b, a, and e reflect the differences in the two phases between which the compound is transferred.

In Equation (1), the relationships between the logBB values and the LFER parameters have been studied. Analyzing the R^2^ values, it can be concluded that there is a good correlation between the logBB values and the LFER parameters. However, taking into account some variance parameters (Table 5), it turns out that Equation (1) requires improvements.

The standard error of a coefficient (SE coefficient) measures the precision of the estimation of an unknown value of a given coefficient, and, in our case, this is logBB. The standard errors of the LFER coefficients are similar to each other. The only exception to this is the McGowan volume. Therefore, the model is able to estimate the logBB values with greater precision using V than the other LFER parameters. In the LFER approach, solute properties are characterized by their dipolarity/polarizability, hydrogen-bonding, and the McGowan volume, which is a product of the exoergic solute–solvent dispersion interaction and the endoergic separation of the solvent molecules [76]. Moreover, T-values measure the ratio between the coefficient and the standard error obtained for the A, B, S, and E parameters, which are definitely too small to declare statistical significance here. The resulting *p*-value, being the probability that measures the evidence against the null hypothesis, only in two cases (for B and V) was smaller than the significance level, and it is equal to 0 here. This indicates that there is a statistically significant association between the response variable and the logBB values. However, in the case of the B parameter, the variance inflation factor (VIF) is higher than for the V and A parameters. Nevertheless, all the obtained VIF values were greater than 5, which means that multicollinearity exists in the context of the correlation between predictors. Therefore, many attempts have been made to improve QSAR model for BBB permeation.

According to the data in Table 5, the model based on E, ΔlogP, and logP_ow_ (Equation (2)) has been significantly improved when compared with the former model. The proportion of the total variation that is explained by the regression model (R^2^ values) can be determined by comparing the regression sum of squares (SS regression) to the total sum of squares (SS total). In both cases, the R^2^ values were similar. However, the obtained F-values indicate that, in Equation (2), all the variables are associated with the calculated logBB values, unlike Equation (1), in which only the B and V parameters showed F-values higher than 1. In each case, the lowest value of the adjusted mean square error of leave-ten-out cross-validation (adjusted MSECV) determined the choice between the training and the test sets. In the case of the first QSAR model, the value of the adjusted MSECV was equal to 0.2335, and it was 0.1905 for the second model. Some of statistical parameters of the leave-ten-out cross-validation are shown in Table 6.

The calculated SE coefficient values obtained for Equation (2) have been significantly reduced in comparison to those in Equation (1). Moreover, the T-values and *p*-values have been considerably improved, e.g., the *p*-values in Equation (2) are in each case smaller than 0.05, which suggests that the differences between the variables are statistically significant, which is also confirmed by the much lower VIF values than those in Equation (1). Consequently, both the predictability and reliability of the QSAR model expressed in Equation (2) have been proved. Therefore, it should be emphasized that the model based on E, ΔlogP, and logP_ow_ is relevant for describing the BBB permeability of the studied compounds.

#### 3.1.2. BBB Descriptors Calculated in Silico

The permeability–surface area product (PS) is a complex parameter because it encompasses passive transcellular diffusion across the BBB as well as the possible contribution of active transport. In fact, it measures the permeability of the BBB to contrast material. Small lipophilic agents cross the endothelial cell membrane by passive diffusion [7]. The diffusion flux of contrast agents across the capillary endothelium is dependent on both the diffusion coefficient and the total surface area of the pores [77].

Permeability is related to the diffusion coefficient of the contrast agent in the assumed water-filled capillary endothelium. It is expressed as follows: PS = - CBFln(1-E_Fr_), where CBF is the cerebral blood flow and E_Fr_ is the extraction fraction, that is, the fraction of contrast material that leaks into the extravascular from the intravascular space [78]. Moreover, the rate of brain penetration (logPS) is also defined from the kinetic equation of capillary transport: PS = −F(1 − e^−K^_in_^/F^). It is then equal to the influx rate constant (K_in_), corrected for the blood flow rate in the cerebral microcapillaries, denoted as F.

The other obtained value is the brain/plasma equilibration rate, given by log(PS_Fubrain_), which is a combination of the permeation rate and the fraction unbound in brain. A very important parameter is also the fraction unbound in plasma (F_u_), which is measured using the equilibrium dialysis.

Compounds I–IV demonstrated similar log(PS_Fubrain_) values (with an average equal to −4.85) in contrast to the compounds not exceeding the BBB (V–VII, with an average equal to −6.8). Saponins I–IV, as well as saponins V–VII, probably give a similar time to reach brain equilibrium. Simultaneously, analyzing the values of the fraction unbound in brain, it can be concluded that among the saponins crossing the BBB, the longest time to achieve brain equilibrium will be required by AII and the shortest by AI. Confirmation of this is also given by the values of the fraction unbound in plasma (which were highest for AII and lowest for AI). However, it is often desirable in CNS drug discovery to find substances that can quickly penetrate the brain, whereby the unbound drug concentration at the targeted site in the brain tissue can reach the plasma unbound concentration quickly after administration [51].

### 3.2. Recovery of Astragaloside IV from Plant Material

Semi-preparative HPLC chromatography was selected to deliver high purity saponin, even if the isolation was performed via a time-consuming protocol. In the former studies, not much can be found about the actual isolation of astragaloside IV. The majority of manuscripts describe the results of the pharmacological activity that astragaloside IV has exhibited in a living organism, where astragaloside IV was obtained from a purchased standard compound [79]. The previous report of Lee and co-investigators confirmed the possibility to isolate astragaloside II from *Astragalus membranaceus* using a flash chromatograph, which certainly shortened the time of separation; however, astragaloside IV was not within the obtained compounds [80], contrary to Lai and colleagues who isolated this saponin in a bioactivity guided fractionation model [81]. For this purpose, the authors prepared a series of chromatographic columns and tried to precipitate the desired compounds to fractionate a water extract obtained from *A. membranacaeus*. For the isolation of astragaloside IV, the authors first eluted a chromatographic column with a mixture of ethyl acetate and methanol (8:2); (6:4); (4:6) *v*/*v*, pure methanol, and 70% methanol. The obtained fractions were purified with the Sephadex LH-20 with 40% methanol, then passed through a chromatographic column (silica gel) with MeOH (10–60%), and finally precipitated with acetone to deliver astragaloside IV. The herein applied methodology was far simpler, but the quantity of runs that needed to be done prior to the in vivo tests on mice was overwhelming.

### 3.3. In Vivo and Postmortem Studies

Blood–brain barrier permeation studies, especially for not completely investigated substances, are very important in the perspective of the possibility of their further use in the treatment of neurodegenerative diseases. As shown by previous research, astragaloside IV in doses of 10 and 20 mg/kg b.w. in rats can significantly weaken the permeability of the blood–brain barrier in comparison with a vehicle group after an ischemia–reperfusion injury [82]. The protective effects of AIV against an ischemia–reperfusion injury have also been identified in a murine model of transient focal ischemia related to antioxidation. Therefore, astragaloside IV has been recognized as a promising agent for treating apoplexy [83,84,85]. Moreover, it has been found that AIV can relieve the decrease in the level of dopamine in 6-hydroxydopamine-induced substantia neurons, and, therefore, it can be used in the treatment of Parkinson’s disease [86].

In this investigation, all planned studies were carried out to show the path from in silico modeling to the postmortem determination of the concentration of the most neuroactive component of the tested milkvetch roots in both the brain tissues and blood of mice. Astragalosides I–VII were used for in silico studies, whereas the most active saponin (astragaloside IV) was first isolated from the tested extract by semi-preparative HPLC chromatography and then employed for the postmortem studies.

Moreover, the highest activity of astragaloside IV was proven during the in vitro acetylcholinesterase inhibition activity test. The chromatogram allowed the selection of AIV as the most neuroactive saponin among the tested saponins. Based on these in vitro and in silico results, the tests on animals could be planned. As a result, it can be clearly seen that the introductory of in silico studies has been confirmed by the animal experiments (Figure 7). Both the experimentally obtained and in silico calculated logBB values prove that astragaloside IV is able to cross the blood–brain barrier. According to the obtained results, the actual concentration of astragaloside IV in brain tissues was approximately three times lower than that in the mice plasma, thus it gives a clear view that this saponin is able to affect the physiology of the CNS system.

In the scientific literature, we can find some examples of careful studies on the penetration of saponins across the blood–brain barrier. The results differ—depending on the structure of the studied compounds. In the studies of Feng et al. [87], the saponins from *Polygalae radix*, which belong to the group of triterpene saponins, e.g., desacylsenegasaponin B, were found to not be able to penetrate the BBB. None from the seven saponins’ metabolites present in the plasma were detected in the brains of rats. On the other hand, anemoside B4 and anemoside A3, which belong to the lupane-type group of triterpenoid saponins, present in the extracts of the *Pulsatilla* species, were proven to cross the BBB [88]. In the studies of Tian and co-workers on *Gouteng-Baitouweng,* an herbal composition composed of *Uncaria rhynchophylla* and *Pulsatilla chinensis* organs, the maximal concentrations of anemosides B4 and A3 were calculated as 187.2 ± 83.3 and 17.8 ± 6.9 ng/g, respectively, after the oral administration of a decoction (25 g/kg) in rats. The systemic plasma content obtained for both compounds was equal to 3180.6 ± 828.5 and 76.8 ± 28.2 ng/kg, respectively. In the light of our findings, the content of anemoside A3 in the plasma in relation to the brain tissue concentration was similar. The ratio was around 4:1 in the studies of Tian and co-investigators.

## 4. Materials and Methods

### 4.1. Computer Programs

Within in silico studies, ACD/Percepta software (version 2012, Advanced Chemistry Development, Inc., Toronto, ON, Canada) was used. Statistical analysis of the obtained results was made using the Minitab 18 Statistical Software (Minitab Inc., State College, PA, USA). For studying the TLC-bioautography chromatogram, ImageJ software (1.48v, Wayne Rasband, National Institutes of Health, Maryland, USA) was used.

### 4.2. Extraction of Plant Material

The roots of *Astragalus mongholicus* were obtained from Ulaanbaatar (Bayangol district) in July 2017. They were authenticated by Dr. Otgonbataar Urjin from the Mongolian National University of Medical Sciences. The extraction process was performed on dried and ground roots. In the process, 500 g of powdered material was extracted by a mixture of water/methanol (50:50 *v*/*v*) as a result of overnight maceration. The solid/liquid ratio was equal to 1:10.

### 4.3. Determination of the Acetylcholinesterase Inhibitory Activity in a TLC-Bioautography Based Assay

To evaluate the blood–brain barrier permeability of the extract constituents, an acetylcholinesterase (AChE) inhibitory activity assay was performed for the major constituents of the tested extract. For this purpose, the solutions of analytical standards of astragalosides I, III, and IV (Sigma Aldrich, St. Louis, MO, USA) were prepared in methanol (Merck, Darmstadt, Germany, p.a.) at a concentration of 1 mg/mL. Next, 4 μL of all standards was introduced onto the TLC plates (silica gel, F_254s_, 10 × 10 mm, Merck, Darmstadt, Germany) with the CAMAG Linomat 5 instrument (Camag, Muttenz, Switzerland) as 8-mm bands. The TLC plates were further developed in horizontal TLC sandwich chambers (Chromdes, Lublin, Poland) at a humidity rate of 75% after the initial saturation of the chamber with the developing phase. The mobile phase was composed of water/methanol/ethyl acetate (1.7:2.1:16.2 *v*/*v*/*v*; Merck, p.a) with the addition of 30 mg of naphthyl acetate (Sigma Aldrich, Saint Louis, MO, USA; p.a) per 20 mL of the mobile phase and 2% ammonia (POCH, Gliwice, Poland, p.a.). The further stages of the study were conducted in accordance with the procedure described by Kukula-Koch and Mroczek [59]. Briefly, the developed TLC plates were first dried in the air, sprayed with the solution of the acetylcholinesterase enzyme in the bovine serum, and incubated for 10 min at 37 °C in an increased humidity environment. The chromatograms were left under the fume hood and after 10 min they were derivatized with a solution of Fast Blue B salt. The following reagents were used in these steps (Sigma Aldrich): acetylcholinesterase (type VI-S), bovine serum albumin (> 96%), Fast Blue B (95%), and 2-amino-2-(hydroxymethyl)-1,3-propanediol (> 99.9%). The detection and visualization of the obtained chromatograms was done under UV light at a wavelength of 254 nm and was then archived using a Reprostar 3 video camera with the WinCats software (version 1.4.4, Camag, Muttenz, Switzerland). The chromatogram was carefully studied with the help of an image processing program (ImageJ software; 1.48v, Wayne Rasband, National Institutes of Health, Maryland, USA) and the peak areas were recorded for the studied astragalosides. The spots were comparable as each compound was applied at a concentration of 1 mg/mL (4 μL) at a band of 8 mm.

### 4.4. Isolation of the Selected Active Natural Product

Semi-preparative HPLC was used to isolate and purify the most active saponin in the extract. For this purpose, the Vp liquid chromatographic system was used equipped with an LC 10AT pump, an SPD 10A UV-VIS detector, an SCL 10A system controller, a CTO-10 AS chromatographic oven, and a Rheodyne injector valve with 20 μL loop (Shimadzu, Kyoto, Japan). This system utilizes the Class-Vp computer program to control the hardware and acquire and store data, as well as determine retention times. The analysis was carried out at a wavelength of 254 nm and at a temperature of 20 °C.

The isolation of the individual components was carried out with the Kromasil 100-10-C-18 column (250 mm × 4.6 mm, 10 μm; Akzo Nobel, Bohus, Sweden) and a mobile phase composed of acetonitrile and water. The elution was performed using a 0.5 mL/min mobile phase gradient programmed from 90% water (A) to 60% acetonitrile (B) as follows (A:B): 90:10 (t = 5 min), 80:20 (t = 10 min), 75:25 (t = 20 min), 67:33 (t = 30 min), 65:35 (t = 40 min), 40:60 (t = 55min). Overall, 52 fractions were collected in one cycle of the separation process. All experiments were reproduced 10 times. The compositions of the obtained fractions were evaluated using liquid chromatography mass spectrometry, specifically, HPLC/DAD/ESI-Q-TOF-MS (Agilent Technologies, Santa Clara, CA, USA).

The performed optimization of the mass spectrometer settings led to the selection of the following parameters as the most advantageous ones, namely a capillary voltage of 3500 V, a fragmentor voltage of 110 V, and a gas temperature of 325 °C. The other parameters (i.e., nozzle voltage or skimmer voltage) did not have a significant influence on the instrument response in the analysis of astragaloside IV. Here, 10 V for the CID fragmentation energy was found to be preferable to compare the MS/MS spectra of the standard and the biological samples; however, the fragmentation of the investigated saponin was very strong.

### 4.5. Postmortem Studies

The performed tests were first approved by the Ethics Committee at the University of Life Sciences in Lublin, Poland (26 Mar 2018; agreement number: 45/2018).

Mice (Swiss Webster, male, 36 subjects) were treated with astragaloside IV (12.5 and 25 mg/kg b.w.), isolated from the tested aqueous methanolic extract of *Astragalus mongholicus* roots. The relevant fractions of the extract rich in AIV were collected together and evaporated to dryness under reduced pressure at 40 °C. The isolated AIV was redissolved in saline and then used in the in vivo studies Astragaloside IV was given by a single intraperitoneal administration (i.p.) for each of the tested animals. Out of the studied animals, the authors analyzed the content of astragaloside IV in 6 brains from each of the two groups that were dosed at 12.5 and 25 mg/kg.

The doses of astragaloside were chosen based on the literature data [89], as well as preliminary studies. The blood samples collected from the animals delivered plasma that was immediately deep frozen and kept for quantitative analysis. Then, the mice were decapitated 1 h after the last injection and the brains were collected and homogenized in a plastic Eppendorff vial kept on ice. The homogenates of the brain and plasma samples were further vortexed for 10 min with 200 μL of 50% ethanol. The Eppendorf vials were further centrifuged at 3500 RPM for 5 min and the supernatant was filtered through a nylon syringe filter with a diameter of 0.22 μm to the autosampler vials with glass inserts.

The analytical standard of astragaloside IV was dissolved in 50% ethanol and dissolved into 10 different concentrations within the range of 0.002–0.2 mg/mL to obtain the calibration curve. Due to the low sizes and masses of brains, astragaloside IV was quantified in these organs after the addition of the reference compound to the samples. For this purpose, 100 µL of 0.5 mg/mL astragaloside IV was introduced to each brain sample. The peak areas of astragaloside IV in the plasma and brain samples were compared to the blank solution of a standard compound.

### 4.6. HPLC-MS Based Qualitative and Quantitative Analyses of Biological Samples

The qualitative and quantitative analyses of the plant extract, obtained fractions, purified compounds, biological samples, and the analytical standards were performed using an Agilent Technologies (Santa Clara, CA, USA) mass spectrometer (ESI-Q-TOF-MS, 6500 Series) with a high performance liquid chromatograph (HPLC), also by Agilent Technologies (1200 Series, Santa Clara, CA, USA), equipped with a binary pump, solvent degasser, thermostated column chamber, autosampler, and a PDA detector. The analyses were performed using spectroscopic purity solvents (acetonitrile, water, and formic acid; J.T. Baker).

The exact mass spectrometer was carefully adjusted on the astragaloside IV standard sample to induce the highest possible response from the detector. For this purpose, a series of astragaloside IV injections was made with a capillary voltage from 2000 to 4000 V (2000, 2500, 3000, 3500, and 4000 V), a fragmentor voltage from 90 to 130 V (90, 100, 110, 120, and 130 V), and a gas temperature from 225 to 325 °C (225, 250, 275, 300, and 325 °C). The skimmer voltage was set at 65 V, the nebulizer at 30 PSI, the gas flow at 12 L/h, and the operation mode to the positive ionization mode. The spectra were collected between 100 and 1200 *m*/*z*, and the injection volume was set at 20 μL. The two values of the CID energy (10 and 20 V) were applied to obtain the MS/MS spectra. The column temperature was 20 °C and the following gradient of acetonitrile with the addition of 0.2% formic acid (solvent B) in 0.2% formic acid (solvent A) was applied: 0 min 2% B in A; 2 min 10% B in A; 6 min 40% B in A; 35 min 95% B in A, held for 1 min; 37 min 2% of B in A. The analysis lasted for 40 min, the post run was set at 10 min, and the applied flow rate was equal to 0.2 mL/min. The Zorbax RP18 column by Agilent Technologies (Santa Clara, CA, USA) was selected for the analysis (150 mm × 2.1. mm, d = 3.5 μm).

## 5. Conclusions

In this study, triterpenoid saponins from the root of *Astragalus mongholicus* were tested. This root is commonly used in the traditional medicine of China and currently is among the 10 most used plant medicines in the world, especially in the treatment of cancer [90]. Even if frequently administered, *Astragalus* is mainly supplemented in the form of the total extract. Several previous studies have already mentioned the high potential of *Astragalus* in the elevation of memory and cognition; however, not much is known about the activity of single metabolites of the plant. No experimental values of logBB have yet been found in the literature.

The aim of this study was to evaluate the ability to cross the BBB by triterpenoid saponins occurring in the *Astragali radix* based on a QSAR methodology combined with postmortem studies on the brain tissues of mice. The paper shows the results of the analytical studies, which include in silico BBB tests with QSAR modeling, an in vitro acetylcholinesterase (AChE) inhibitory assay, and postmortem studies of the determination of the concentration of the most neuroactive compound in the brain tissues and blood of mice. Based on the obtained values, the logBB value was determined experimentally.

The presented computational studies confirm the ability of saponins I–IV to cross the blood–brain barrier. Statistical analyses of the newly constructed model based on E, ΔlogP, and logP_ow_ has proven both the applicability and predictability in estimating the ability of triterpenoid saponins to cross the BBB. In the in vitro tests, the most neuroactive compound of the tested compounds was indicated, although all saponins inhibited AChE. Astragaloside IV was then successfully isolated using semi-preparative HPLC.

Consequently, it was appropriate to investigate whether the most neuroactive compound of the root of *Astragalus* could be located in the brain. The results of postmortem studies show a regular dependence of the final concentration of AIV in the analyzed brain samples of animals treated with 12.5 and 25 mg/kg b.w. (0.00012299 and 0.0002306 mg, respectively, per one brain). The accumulation of the active substance in the brain tissues confirms that it can cross the blood–brain barrier. The experimentally determined logBB value was equal to 0.49 ± 0.03. The logBB value calculated on the basis of Equation (2) is similar to that determined experimentally with the mice brain tissues (the approximation error is equal to 0.056 here). This means that the newly established model corresponds to the value obtained in vivo.

## Figures and Tables

**Table 1:** Previously established quantitative structure–activity relationship (QSAR) models for estimation of ability of compounds to cross the blood–brain barrier (BBB).

References	Descriptor Variables	Number of Compounds	Number of Descriptor Variables	R^2^	Standard Error
Platts et al. [23]	Linear free energy relationship (LFER) parameters (see Section 3.1.1.)	148	5	0.71	0.367
Young et al. [24]	Molecular weight (MW)	6	1	0.797	0.760
Young et al. [24]	The difference between the *n*-octanol/water and cyclohexane/water logP values (ΔlogP)	6	1	0.98	0.249
Rose et al. [25]	The hydrogen electrotopological index for hydrogen bond donors, the second order difference valence molecular connectivity index, and the hydrogen E-state index for aromatic CHs groups	102	3	0.66	0.45
van de Waterbeemd and Kansy [26]	Polarity	56	1	0.939	0.431
van de Waterbeemd and Kansy [26]	Polar surface area (PSA)	56	1	0.972	0.294
Lombardo et al. [27]	Solvation free energy in water	55	1	0.82	0.41
Kaliszan and Markuszewski [28]	MW and logarithm of n-octanol/water partition coefficient (logP_ow_)	20	2	0.801	0.486
Kaliszan and Markuszewski [28]	MW and logarithm of cyclohexane/water partition coefficient (logP_cw_)	20	2	0.919	0.321
Salminen et al. [29]	MW, indicator variable of ionization, and logP_o/w_	26	3	0.839	0.47
Clark [30]	PSA and ClogP	55	2	0.887	0.354
Feher et al. [31]	The number of hydrogen-bond acceptors, logP, and PSA	61	3	0.730	0.424
Keseru and Molnar [32]	Free energy of solvation	55	1	0.85	0.37
Kaznessis et al. [33]	Solvent accessible surface area, solute dipole, number of hydrogen-bond acceptors and donors, and molecular volume	76	5	0.97	0.173
Narayanan and Gunturi [34]	Variable selection and modeling method based on the prediction (VSMP) used for the selection of descriptor combinations based on two parameters	88	324		
This paper	LFER parameters	40	5	0.768	0.432
This paper	Excess molar refraction (E), ΔlogP, and logP_o/w_	40	3	0.740	0.436

**Table 2:** The chemical structures of the tested compounds.

Name	Structure
Astragaloside I (AI)	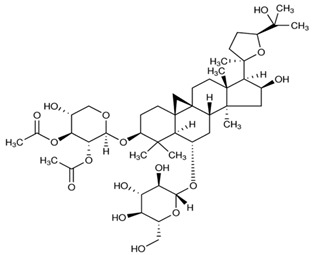
Astragaloside II (AII)	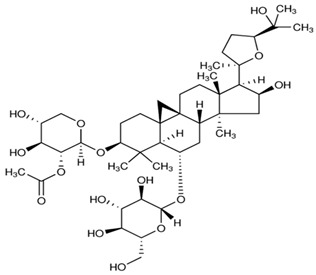
Astragaloside III (AIII)	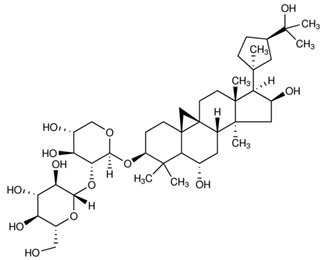
Astragaloside IV (AIV)	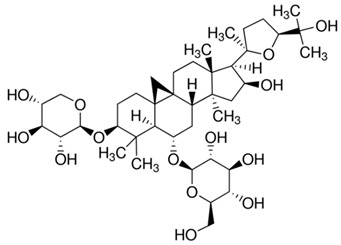
Astragaloside V (AV)	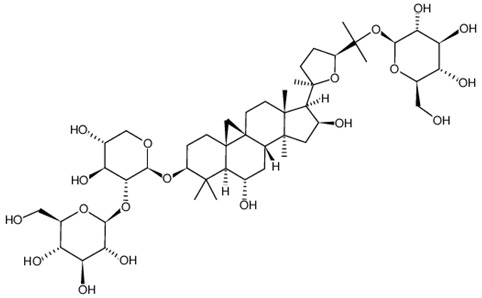
Astragaloside VI (AVI)	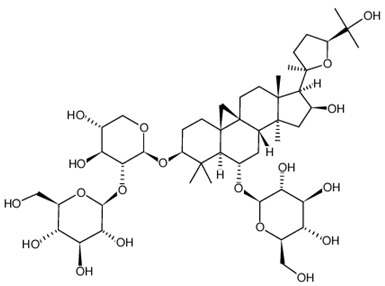
Astragaloside VII (AVII)	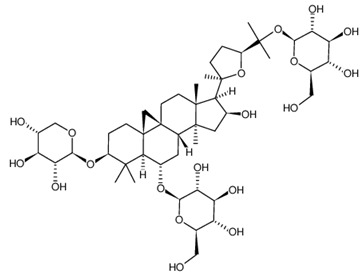

**Table 3:** The BBB descriptors calculated in silico (ACD/Percepta). logBB, Blood–brain barrier penetration descriptor; logPS, Logarithmic permeability–surface area product; log(PS_Fubrain_), Brain/plasma equilibration rate; F_u_, Fraction unbound in plasma; F_b_, fraction unbound in the brain.

No.	CAS No.	logBB	logPS	log(PS_Fubrain_)	F_u_	F_b_
1	84680-75-1	0.46	−4.0	−4.6	0.67	0.24
2	84676-89-1	0.11	−4.7	−4.9	0.80	0.62
3	84687-42-3	0.15	−4.7	−5.0	0.78	0.56
4	84687-43-4	0.24	−4.6	−4.9	0.79	0.45
5	84687-44-5	<−2	<−5	−6.9	0.87	0.96
6	84687-45-6	<−2	<−5	−6.8	0.88	0.94
7	84687-46-7	<−2	<−5	−6.7	0.88	0.94

**Table 4:** The LFER and chosen physicochemical parameters, calculated for the entire set of the tested compounds (ACD/Percepta). A, B, S, E, V, LFER parameters; MW, Molecular weight; TPSA, Topological polar surface area; logP_ow_, Logarithm of the *n*-octanol/water partition coefficient; logP_cw_, Logarithm of the cyclohexane/water partition coefficient; logP_hw_, Logarithm of heptane/water partition coefficient; ΔlogP, difference between the *n*-octanol/water and cyclohexane/water logP values.

No.	CAS No.	A	B	S	E	V	MW	TPSA	logP_ow_	Polarizability	logP_cw_	logP_hw_	ΔlogP
1	84680-75-1	1.72	4.87	4.62	3.72	6.4335	869.04	240.36	5.02	86.01	−7.125	−7.543	12.145
2	84676-89-1	1.99	4.73	4.57	3.86	5.8542	798.95	234.29	3.47	78.53	−8.445	−8.9	11.915
3	84687-42-3	2.33	4.69	4.4	3.97	5.8385	784.97	228.22	3.767	78.41	−9.269	−9.533	13.036
4	84687-43-4	2.26	4.71	4.38	3.98	5.8385	784.97	228.22	3.757	78.41	−9.015	−9.3	12.772
5	84687-44-5	3.09	6.12	5.52	4.92	6.8688	947.11	307.37	2.093	91.78	−15.628	−15.861	17.721
6	84687-45-6	3.01	6.22	5.46	4.96	6.8688	947.11	307.37	1.899	91.78	−15.592	−15.823	17.491
7	84687-46-7	3.03	6.15	5.5	4.93	6.8688	947.11	307.37	2.083	91.78	−15.412	−15.661	17.495
8	30516-87-1	0.47	1.7	1.77	1.62	1.8192	267.24	109.19	0.171	0	−3.401	−3.625	3.572
9	73590-58-6	0.35	2.05	3.18	2.67	2.5161	345.42	96.31	1.444	37.27	−1.995	−2.926	3.439
10	101-40-6	0.13	0.53	0.43	0.37	1.5088	155.28	12.03	3.733	19.75	3.485	3.554	0.248
11	23830-88-8	0.42	1.04	1.14	1.5	1.5317	230.09	36.42	1.987	22.71	−0.264	−0.376	2.251
12	103-90-2	0.91	0.93	1.66	1.12	1.1724	151.16	49.33	0.149	16.81	−4.518	−4.582	4.667
13	60-80-0	0	1.28	1.75	1.42	1.4846	188.23	23.55	0.24	21.63	−1.228	−1.691	1.468
14	54910-89-3	0.13	0.78	1.19	1.01	2.24	309.33	21.26	5.147	31.67	4.728	4.46	0.419
15	54739-18-3	0.23	1.14	0.95	0.66	2.3113	318.33	56.84	4.267	30.46	3.083	3.056	1.184
16	79559-97-0	0.13	0.67	1.44	1.83	2.2647	306.23	12.03	5.888	34.02	5.705	5.222	0.183
17	53179-11-6	0.31	1.88	2.9	2.76	3.7697	477.04	43.78	6.431	54.52	4.235	3.349	2.196
18	7481-89-2	0.44	1.9	1.78	1.63	1.506	211.22	88.15	−1.653	20.19	−5.618	−5.815	3.965
19	161814-49-9	0.64	2.61	3.52	2.71	3.8194	505.63	139.57	3.321	53.37	−1.597	−2.428	4.918
20	69655-05-6	0.31	1.77	1.85	2.03	1.5951	236.23	88.74	−0.716	22.67	−3.887	−4.24	3.171
21	129618-40-2	0.42	1.37	2.29	2.36	1.9446	266.3	58.12	1.83	29.23	−1.059	−1.667	2.889
22	159989-64-7	1.27	2.81	3.62	3.2	4.5367	567.78	127.2	5.66	64.37	−1.292	−1.941	6.952
23	151-83-7	0.24	1.42	1.55	1.27	2.0903	262.3	66.48	2.201	27.68	0.175	−0.087	2.026
24	76-73-3	0.52	1.3	1.41	1.11	1.8945	238.28	75.27	1.971	24.68	−1.018	−1.108	2.989
25	76-75-5	0.51	1.34	2	1.49	1.9014	242.34	90.29	1.45	25.99	−1.838	−2.195	3.288
26	59468-90-5	0.31	1.21	1.96	2.64	2.3215	341.77	49.89	4.219	35.95	2.491	1.893	1.728
27	1088-11-5	0.47	0.88	1.94	2.05	1.933	270.71	41.46	3.435	29.97	1.115	0.616	2.320
28	439-14-5	0	0.94	1.95	2.07	2.0739	284.74	32.67	3.784	32.08	3.27	2.571	0.514
29	13655-52-2	0.29	1.36	1.12	1.18	2.1587	249.35	41.49	3.143	29.75	1.346	1.263	1.797
30	29122-68-7	0.78	1.85	1.97	1.48	2.1763	266.34	84.58	0.839	29.44	−3.963	−4.105	4.802
31	63659-18-7	0.29	1.53	1.31	1.31	2.5745	307.43	50.72	3.979	35.25	2.145	1.995	1.834
32	120014-06-4	0	1.5	2.49	2.12	3.0307	379.49	38.77	5.024	43.77	4.121	3.282	0.903
33	357-70-0	0.31	1.45	1.92	1.89	2.1734	287.35	41.93	2.375	31.84	0.011	−0.431	2.364
34	123441-03-2	0	1.23	1.47	1.05	2.1176	250.34	32.78	2.915	28.99	2.096	1.75	0.819
35	142852-50-4	0.13	1.3	2.12	2.19	3.154	376.53	32.34	6.551	45.53	5.762	5.073	0.789
36	91374-21-9	0.41	1.27	1.41	1.38	2.2321	260.37	32.34	3.51	31.07	1.298	1.113	2.212
37	52-26-6	0.5	1.47	1.59	2.23	2.0648	285.34	52.93	2.471	30.93	−0.426	−0.694	2.897
38	83903-06-4	0.4	2.57	2.2	2.55	3.1776	413.54	108.06	2.512	46.52	−1.054	−1.461	3.566
39	59-33-6	0	1.45	1.73	1.66	2.3868	285.38	28.6	3.353	34.67	2.433	1.947	0.920
40	83-67-0	0.24	1.22	1.89	1.46	1.2223	180.16	67.23	−0.635	17.86	−3.184	−3.61	2.549
41	36318-56-6	0.42	1.13	0.90	1.26	1.4278	175.23	36.42	1.398	20.82	−0.969	−0.941	2.367
42	4201-26-7	0.42	1.11	1.01	1.41	1.5502	209.68	36.42	1.902	22.64	−0.377	−0.416	2.279
43	38941-33-2	0.42	1.01	1.46	2.17	1.8119	397.89	36.42	3.199	28.05	1.154	0.828	2.045
44	76-57-3	0.23	1.58	1.92	2.16	2.2057	299.36	41.93	2.232	32.85	0.078	−0.405	2.154
45	66357-35-5	0.13	1.60	1.40	1.43	2.3985	314.4	114.9	3.067	33.41	1.586	1.337	1.481
46	82626-48-0	0.00	1.33	2.39	2.35	2.4740	307.39	38.13	3.793	37.08	2.857	2.013	0.936
47	133099-04-4	0.49	1.58	2.82	2.80	3.3978	426.55	55.56	6.129	50.08	3.455	2.627	2.674

**Table 5:** Analysis of variance obtained for Equations (1) and (2). SE Coefficient, standard errors; Adjusted SS, adjusted sum of squares; Adjusted MSE, adjusted mean square errors.

**Descriptors**	**SE Coefficient**	**T-Value**	***p*-Value**	**Variance Inflation Factors (VIF)**	**Adjusted SS**	**Adjusted MSE**	**F-Value**
**Regression Equation (1)**					23.6018	4.7204	20.22
A	0.289	−0.38	0.706	10.97	0.0337	0.0337	0.14
B	0.255	−4.61	0.000	27.95	4.9542	4.9542	21.22
S	0.283	−0.62	0.538	24.38	0.0905	0.0905	0.39
E	0.256	−0.95	0.351	15.20	0.2086	0.2086	0.89
V	0.158	7.58	0.000	12.43	13.4293	13.4293	57.52
**Error**					7.9384	0.2335	
**Total**					31.5402		
**Descriptors**	**SE Coefficient**	**T-Value**	***p*-Value**	**VIF**	**Adjusted SS**	**Adjusted MSE**	**F-Value**
**Regression Equation (2)**					24.6816	8.2272	43.18
E	0.105	−2.08	0.045	3.13	0.8221	0.8221	4.31
ΔlogP	0.0371	−2.64	0.012	3.20	1.3298	1.3298	6.98
logP_ow_	0.0412	6.74	0.000	1.32	8.6603	8.6603	45.46
**Error**					6.8586	0.1905	
**Total**					31.5402		

**Table 6:** Statistical parameters obtained for the newly established models. N, number of compounds; R^2^, coefficients of determination; RMSE, root-mean-square error of calibration; RMSECV, root-mean-square error of leave-ten-out cross-validation; PRESS, predicted residual sum of squares.

QSAR Model	Statistical Parameters	Training Set	Test Set	External Validation
1	N	30	10	7
	R^2^	82.43%	80.80%	81.01%
	RMSE	0.1797	0.1869	0.4426
	RMSECV	0.4843	-	-
	PRESS	6.5141	9.7206	9.3462
2	N	30	10	7
	R^2^	78.25%	74.12%	79.28%
	RMSE	0.1858	0.2731	0.2340
	RMSECV	0.6819	-	-
	PRESS	8.7754	9.7206	7.5450

## Supplementary Materials

Supplementary materials can be found at https://www.mdpi.com/1422-0067/21/7/2534/s1.

## Abbreviations

AChE	acetylcholinesterase
AD	applicability domain
Adjusted MSE	adjusted mean square errors
Adjusted MSECV	adjusted mean square errors of leave-ten-out cross-validation
Adjusted SS	adjusted sum of squares
AI–AVII	astragalosides I–VII
BBB	blood–brain barrier
CNS	central nervous system
CSF	cerebrospinal fluid barrier
F_b_	fraction unbound in brain
F_u_	fraction unbound in plasma
K_in_	influx rate constant
HPLC	High performance liquid chromatography
LDA	linear discriminant analysis
LFER	Linear free energy relationships
log(PS_Fubrain_)	brain/plasma equilibration rate
logBB	logarithm of the ratio between the brain and the blood concentration
logP_ow_	logarithm of *n*-octanol/water partition coefficient
LTO	leave-ten-out cross-validation
MLR	multiple linear regression
MW	molecular weight
PAMPA	parallel artificial membrane permeability assay
PLS	partial least square
PS	permeability surface area product
PSA	polar surface area
RMSE	root-mean-square error of calibration
RMSECV	root-mean-square error of leave-ten-out cross-validation
PRESS	predicted residual sum of squares
QSAR	quantitative structure–activity relationships
SE coefficient	standard error
SS regression	regression sum of squares
SS total	total sum of squares
VIF	variance inflation factors
