# Supplementary material for: In Silico Studies on Triterpenoid Saponins Permeation through the Blood–Brain Barrier Combined with Postmortem Research on the Brain Tissues of Mice Affected by Astragaloside IV Administration
Source: Int J Mol Sci. 2020 Apr 5;21(7):2534. doi: 10.3390/ijms21072534 (PMC7177733; doi:10.3390/ijms21072534)
Supplement: Supplementary file 1 [file ijms-21-02534-s001.pdf]

Table S1. Experimentally obtained logBB [50] values for the compounds used as the training and test sets in the QSAR studies*.

No.	CAS No.	logBB
1	30516-87-1	-0.72
2	73590-58-6	-0.82
3	101-40-6	1.08
4	23830-88-8	0.16
5	103-90-2	-0.74
6	60-80-0	-0.07
7	54910-89-3	1.08
8	54739-18-3	0.79
9	79559-97-0	1.6
10	53179-11-6	0.77
11	7481-89-2	-1.5
12	161814-49-9	-0.56
13	69655-05-6	-1.28
14	129618-40-2	0
15	159989-64-7	-0.93
16	151-83-7	-0.06
17	76-73-3	0.2
18	76-75-5	-0.45
19	59468-90-5	-0.07
20	1088-11-5	0.61
21	439-14-5	0.56
22	13655-52-2	-0.23
23	29122-68-7	-1
24	63659-18-7	0.39
25	120014-06-4	0.89
26	357-70-0	0.32
27	123441-03-2	0.88
28	142852-50-4	1.14
29	91374-21-9	0.25
30	52-26-6	-0.16
31	83903-06-4	-1.06
32	59-33-6	0.49
33	83-67-0	-0.29
34	36318-56-6	0.04
35	4201-26-7	0.14
36	38941-33-2	0.58
37	76-57-3	0.08
38	66357-35-5	0.32
39	82626-48-0	0.12
40	133099-04-4	0.06
